# The Role of Plasmacytoid Dendritic Cells in the Immune Contexture of *TP53*-Mutated High-Grade Serous Ovarian Cancer

**DOI:** 10.3390/cancers17233877

**Published:** 2025-12-03

**Authors:** Katharina Steger, Heidelinde Fiegl, Katja Rungger, Katharina Leitner, Irina Tsibulak, Barin Feroz, Christoph Ebner, Christian Marth, Hubert Hackl, Alain Gustave Zeimet

**Affiliations:** 1 Department of Obstetrics and Gynecology, Medical University of Innsbruck, 6020 Innsbruck, Austria; katharina.steger@i-med.ac.at (K.S.);; 2 Biocenter, Institute of Bioinformatics, Medical University of Innsbruck, 6020 Innsbruck, Austria

**Keywords:** high-grade serous ovarian cancer, tumor microenvironment, immuno-oncology, dendritic cells, immune landscape, *TP53* mutation

## Abstract

**Background/Objectives**: This study aimed to characterize dendritic cell (DC) heterogeneity, immune associations, and prognostic relevance across three *TP53*-mutated tumor entities—high-grade serous ovarian cancer (HGSOC), triple-negative breast cancer, and endometrial cancer—focusing on plasmacytoid DCs (pDCs) in HGSOC. **Methods**: RNA-sequencing and clinical data of 603 patients from The Cancer Genome Atlas were analyzed. DC subset abundance was assessed for cDC progenitor, conventional DC type 1 (cDC1), conventional DC type 2 (cDC2), plasmacytoid DC (pDC), and mature DC by marker gene signatures. Differences in DC scores across tumors were analyzed using Kruskal–Wallis. Survival analyses were performed using Kaplan–Meier and Cox regression. Spearman’s correlation was used to determine associations between parameters. **Results**: HGSOC showed the lowest pDC abundance, yet high pDC scores were independently associated with shorter PFS (HR = 1.55, 95% CI: 1.05–2.27; *p* = 0.027), representing the only DC-subset-related prognostic signal observed across tumor types. pDCs correlated positively with neutrophils and negatively with monocytes, and pDCs, cDC2s, and cDC progenitors correlated inversely with TMB. No consistent link was found between pDC and *TP53* mutation classes. However, tumors harboring specific *TP53* mutations within established hotspot regions exhibited significantly lower pDC levels (*p* = 0.015). **Conclusions**: Our findings reveal distinct DC infiltration patterns and highlight the immunological vulnerability of *TP53*-mutated HGSOC. pDCs appear to exert a tumor-promoting, immune-evasive role, suggesting that DC function depends on their programming and tumor context. Selective targeting of DC subsets may offer novel therapeutic opportunities in *TP53*-mutated, low-TMB cancers.

## Simple Summary

High-grade serous ovarian cancer (HGSOC), triple-negative breast cancer, and a subset of endometrial cancers frequently share mutations in the *TP53* gene, one of the most frequently mutated tumor suppressor genes across human cancers. Despite this common genetic background, these cancers often do not respond well to modern immunotherapies. Dendritic cells, which act as messengers between the innate and adaptive immune systems, may play a crucial role in shaping the immune environment of such tumors. In this study, we analyzed tumor samples from 603 patients to explore the distribution of different dendritic cell subsets and their relationship to patient outcomes. We found that a specific subset, plasmacytoid dendritic cells, appears to exert a clinically relevant tumor-promoting role in *TP53*-mutated HGSOC, potentially contributing to immune evasion. Selective targeting of dendritic cell subsets could offer novel therapeutic strategies in *TP53*-mutated malignancies with low tumor mutational burden such as HGSOC.

## 1. Introduction

The tumor protein 53 (*TP53*) is the most frequently mutated tumor suppressor gene across all human cancers. It encodes a central tumor-suppressor transcription factor that maintains genomic stability by regulating DNA damage response, cell-cycle arrest, apoptosis, and senescence [1,2]. Loss or mutation of *TP53* leads to impaired DNA repair, chromosomal instability, and uncontrolled proliferation, thereby facilitating malignant transformation [3,4]. Mutations in this gene are a defining feature of several aggressive tumor types, including high-grade serous ovarian cancer (HGSOC), triple-negative breast cancer (TNBC), and a subtype of endometrial cancer (EC) [5]. Despite their common *TP53*-driven genomic instability, these cancers typically exhibit low tumor mutational burden (TMB) and reduced immunogenicity, resulting in limited responses to immune checkpoint inhibitors (ICIs) [6,7,8,9,10,11]. It has been shown that *TP53*-mutated (*TP53mut*) tumors with low TMB exhibit limited T cell infiltration and divergent immune landscapes across tumor types, highlighting the need to better understand the non-lymphoid immune compartments that may shape local immune responses [12,13].

Despite others, these *TP53mut* tumor entities are among the most clinically relevant malignancies in female patients, contributing substantially to global morbidity and mortality [14,15,16]. Their epidemiology is shaped by heterogeneous risk factors—including hereditary predisposition (e.g., mutations in *BRCA1/2,* lynch syndrome), hormonal and reproductive influences, and metabolic factors—and despite multimodal therapy, many patients experience poor outcomes [17,18,19]. Limited responses to ICIs in these low-immunogenic tumors further underscore the need to dissect the innate immune components of their tumor immune microenvironment (TIME) [6,7,8,9,10,11,20].

This study, therefore, builds on our previously published immunogenicity analysis using the same *TP53mut* EC, HGSOC, and TNBC TCGA cohorts [13]. In that work, we examined broader immunogenicity features—such as TMB, interferon (IFN)-signaling, and general immune infiltration patterns. We herein observed that dendritic cell (DC)-related signals showed particularly heterogeneous and entity-specific patterns. These observations motivated a dedicated, cell type-resolved investigation of DC subsets. The present manuscript therefore extends our prior work by focusing specifically on DC heterogeneity, with particular focus on plasmacytoid dendritic cells (pDCs).

HGSOC, in particular, represents a clinically and immunologically challenging tumor entity, characterized not only by frequent *TP53* mutations and low response rates to ICIs, but also by a complex TIME [20,21,22]. Among *TP53mut* tumors, HGSOC is not only most prevalent but also the most lethal malignancy [23]. Thus, it provides a distinct opportunity to investigate mechanisms of immune interplay in a tumor entity with a remarkable immunologically inert setting. Given its clinical relevance, we focused our analyses primarily on *TP53mut* HGSOC. Comparative findings from EC and TNBC are reported to highlight entity-specific differences in the TIME with a special focus on DC heterogeneity. We further aimed to dissect the DC landscape in *TP53mut* HGSOC and to explore its association with immune contexture, TMB, IFN-signaling, and clinical outcome.

As professional antigen-presenting cells, DCs play a pivotal role in coordinating anti-tumor immunity and may critically shape the TIME in low-immunogenic tumors such as HGSOC [24]. However, recent studies revealed profound functional heterogeneity among DC subsets, including conventional DCs type 1 (cDC1), conventional DCs type 2 (cDC2), and pDC, with distinct roles in antigen presentation, T cell activation, but also immune suppression [25].

Among the main DC subsets, cDC1 are the most efficient at cross-presenting tumor antigens to CD8^+^ T cells and are therefore key drivers for the induction of cytotoxic anti-tumor immunity within the TIME [26]. However, they are often less prevalent in low-immunogenic tumors [27]. cDC2s predominantly activate CD4^+^ T cells and direct their differentiation into Th2, Th17, or regulatory T cells depending on the surrounding cytokine milieu, thereby contributing to either immunostimulatory or immunosuppressive responses [28,29]. In contrast, pDCs frequently acquire a tolerogenic phenotype in tumors, characterized by reduced type I IFN, including IFN-α, and production and enhanced expression of immunosuppressive mediators, which can promote regulatory T cell expansion and inhibit effector T cell activity [30,31]. cDC progenitor and immature DCs are recruited into the tumor via chemokines but may remain dysfunctional or fail to mature under the influence of suppressive signals like TGF-β or prostaglandin E2 [32]. In contrast, fully mature DCs (including cDC1 and cDC2) are required for effective migration to lymph nodes and T cell priming; however, these cells are often depleted or functionally impaired in low-immunogenic tumors [31].

The presence, maturation, and function of DCs are tightly regulated by the TIME, which can impair DC-mediated immunity through cytokine-driven tolerogenic reprogramming, metabolic stress, and checkpoint signaling [24]. Moreover, various types of *TP53* mutations, including loss-of-function (LOF) and gain-of-function (GOF), have been implicated in shaping the TIME through altered cytokine and chemokine signaling, thereby, also affecting DC recruitment [33].

This functional specialization and plasticity highlight the importance of in-depth characterization of DC subset composition and activity in *TP53mut* tumors with low immunogenicity. In this context, targeting specific subfractions of DCs—particularly by enhancing their antigen-presenting function or reversing tolerogenic states—has been proposed as a strategy to overcome immunotherapy resistance in these tumors [31,34].

Taken together, these considerations indicate that DC heterogeneity may constitute a key, yet insufficiently explored, determinant of immune variation in *TP53mut* cancers. Therefore, the aim of this study was to systematically characterize DC subset composition across *TP53mut* HGSOC, EC, and TNBC. Particular focus was placed on pDCs in *TP53mut* HGSOC, given the clinical relevance and distinct immunological vulnerability of this entity.

## 2. Materials and Methods

### 2.1. Data Source and Cohort Definition

This study, including 603 female patients, used data from The Cancer Genome Atlas (TCGA) to conduct a retrospective analysis of three cohorts [17,18,19], comprising 158 patients with *TP53mut* EC, 320 patients with *TP53mut* HGSOC, and 125 patients with *TP53mut* TNBC. Somatic mutations in *TP53* and other genes were obtained from the MC3 set of a pan-cancer variant consensus calling from whole exome sequencing of over 10,000 patients and samples from 33 cancer types across various TCGA analyses centers. Seven different variant callers were used, and data were filtered for somatic variants with moderate or high impact [35].

Clinical information and gene expression data (RSEM processed RNA sequencing V2 data) were downloaded from firebrowse.org (Broad Institute). Normalized expression transcript per million (TPM) values were used and log2 transformed with the addition of a pseudocount of 1. Microsatellite instability (MSI) status was retrieved from a study involving gynecological and breast tumors [36].

For all three entities, only tumors harboring at least one somatic *TP53* mutation were included.

TNBC was defined based on immunohistochemical analysis on estrogen receptor (ER-), progesterone receptor (PR-), and HER2 receptor (HER2-), which was complemented by in situ hybridization in cases of equivocal calling. In the EC subgroup, tumors with MSI-H status, *MLH1* mutation, or *POLE* mutation were excluded, as in these, *TP53* mutation could only be a passenger mutation and biology of those subtypes is mainly dependent on *POLE* mutation or MSI-H status. The HGSOC cohort was selected based on grade G3 or G4 serous ovarian carcinomas.

To address the potential influence of *TP53* status on DC profiles, we additionally performed an exploratory post hoc comparison including all available *TP53*-wildtype cases across the three tumor entities.

All data sources used in this study are summarized in Supplementary Table S1.

### 2.2. Functional Classification of TP53 Mutation

To categorize *TP53* mutations into gain-of-function (GOF) and loss-of-function (LOF) groups, we relied exclusively on established, peer-reviewed annotation frameworks. GOF mutations were defined according to Roszkowska et al. [37]. The following mutations were classified as GOF in our analysis: R175H, G245S, R248Q, R248W, R249S, R273C, R273H, and R282W. All other mutations were assigned as LOF according to the above framework.

In addition, functional assignment followed the experimentally validated *TP53* classification provided by Ghosh et al. [38] and the *TP53* database (https://tp53.cancer.gov (accessed on 5 August 2025)), which integrate transcriptional activity, oncogenic behavior, structural impact, and DNA-binding effects.

### 2.3. Dendritic Cell Gene Signature Scoring and TIME Characterization

We used various immune deconvolution approaches including CIBERSORT/LM22, EPIC, MCP-counter, and quanTIseq [39] via the immunedeconv R package (version 2.1.0) [40] to estimate the fractions of tumor infiltrated immune cells from bulk RNA sequencing data. However, these methods do not resolve the biologically distinct DC lineages relevant to this study. Therefore, DC subset abundance was inferred using mean log2 transformed expression values of curated marker genes for the following subsets: cDC1, cDC2, pDC, cDC progenitor, and mature DC. Gene sets were based on the classification in a high-resolution single-cell atlas [41] and are listed in Supplementary Table S2, allowing us to robustly distinguish cDC1, cDC2, pDCs, cDC progenitor states, and mature DC. These signatures have additionally been corroborated in an external HGSOC single-cell dataset [42], supporting their biological specificity. Sample-wise enrichment of selected (hallmark) gene signatures (MSigDB) were analyzed using gene set variation analyses (GSVA) [43].

### 2.4. Statistical Analysis

The non-parametric Mann–Whitney U test or Kruskal–Wallis test was applied to test for statistical significance between two or more groups, respectively, followed by pairwise Dunn’s post hoc test with Bonferroni correction. Correlation analysis was performed using Spearman’s correlation coefficients. Associations between categorical variables, such as pDC expression quartiles and *TP53* mutation classes, were analyzed using Chi-square tests.

For survival analyses, progression-free survival (PFS) was defined as the time from primary diagnosis to histopathological confirmation of recurrence, and overall survival (OS) as the time from diagnosis to death from any cause or last follow-up. Survival analyses were performed using univariate Kaplan–Meier estimates and log-rank tests, with dichotomization at the entity-specific median of the respective DC subset. Multivariable Cox proportional hazards models were used to assess the independent prognostic value of selected variables. Where indicated, additional exploratory analyses were stratified by tumor entity or DC expression quartiles. Significance levels were adjusted using Bonferroni correction.

All reported *p*-values in this manuscript are Bonferroni-adjusted unless explicitly stated otherwise.

Considering the presumed functional differences between the prominent DC subsets, we investigated whether ratios between immune promoting cDC1 and immune suppressing cDC2 and/or pDCs could more accurately reflect prognostic relevance of DCs. Three ratios were calculated from the scores of the DC subsets: cDC1/cDC2, cDC1/pDC, and cDC1/(cDC2 + pDC). The ratios were then dichotomized at the median and subjected to Kaplan–Meier analysis for PFS and OS.

All analyses were performed using SPSS v28.0.0 (IBM) and the statistical software environment R v4.3.1 (R Foundation for Statistical Computing, Vienna, Austria).

### 2.5. Use of Generative AI

Generative AI tools (ChatGPT-5.1 (OpenAI, web-based version accessed on 24 November 2025), DeepL.com (DeepL SE, web-based version accessed on 24 November 2025)) were used to assist in language polishing and layout conceptualization. The scientific content, data analysis, and interpretation were performed entirely by the authors. The Graphical Abstract was created with help of BioRender.com (BioRender, web-based version accessed on 24 November 2025).

## 3. Results

### 3.1. Dendritic Cell Landscape Across TP53-Mutated Tumor Entities

**Figure 1:**
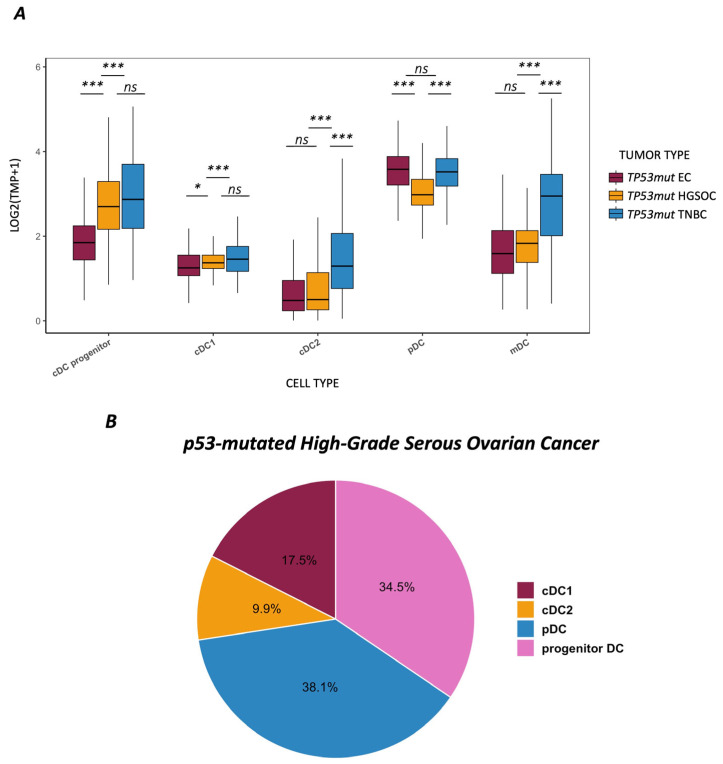
(**A**) Mean expression and distribution of dendritic cell subsets across *TP53*-mutated high-grade serous ovarian cancer (*n* = 320), endometrial cancer (*n* = 158), and triple-negative breast cancer (*n* = 125). Mean expression values (log2 (TPM+1)) of five DC subsets (cDC1, cDC2, pDC, cDC progenitor, and mature DCs) derived from curated marker-gene signatures based on RNA-sequencing data. *p*-values from pairwise Dunn’s post hoc tests with Bonferroni correction are shown (*** *p* < 0.001; * *p* < 0.05; ns, not significant). (**B**) Mean expression and distribution of dendritic cell subsets across *TP53*-mutated high-grade serous ovarian cancer (*n* = 320). Pie chart showing the relative distribution of dendritic cell subsets (cDC1, cDC2, pDC, and cDC progenitor) in HGSOC. Mature DCs are not depicted separately and accounted for 18.2% of the overall DCs.

DC subset signatures varied significantly across the three *TP53mut* tumor entities HGSOC, EC, and TNBC (*p* < 0.001; see Figure 1A). Compared to EC, both other tumor entities exhibited higher cDC progenitor and cDC1 (*p* < 0.001 and *p* = 0.028, respectively). TNBC expressed the highest cDC2 and mature DC scores (*p* < 0.001) as compared to the other two tumor types. Of special note is that pDCs were by far lowest in HGSOC compared to the TIME of the two other *TP53mut* entities, EC and TNBC (*p* < 0.001). The distinct DC profile of HGSOC as depicted in Figure 1B indicates that cDC1 and cDC2 are the most abundant subsets. cDC progenitor and mature DCs showed moderate expression but pDCs were less represented comparatively.

### 3.2. Interferon Signaling and Immune Correlations in HGSOC

Interestingly, only mature DCs exhibited a negative correlation with the IFN-α response score surrogating activation of innate immunity, which as such is frequently linked to tumor promotion (r = −0.238, *p* = 0.024). This is in line with the revealed positive association of IFN-α response and abundance of M2-macrophage TIME infiltration in HGSOC (r = 0.207, *p* = 0.014). However, no other DC subsets were associated with activity of either IFN-α or IFN-γ signaling. pDCs were negatively correlated with monocyte abundance (r = −0.209, *p* = 0.007) and positively correlated with neutrophils (r = 0.171, *p* = 0.028). No associations between the various DC subsets and other immune cell populations (such as T cells, B cells, NK cells, and especially Tregs, etc.) present in the TIME of HGSOC were disclosed. All correlation coefficients and *p*-values reported in this section are summarized in Supplementary Table S3.

### 3.3. Prognostic Relevance of pDCs in HGSOC

**Figure 2:**
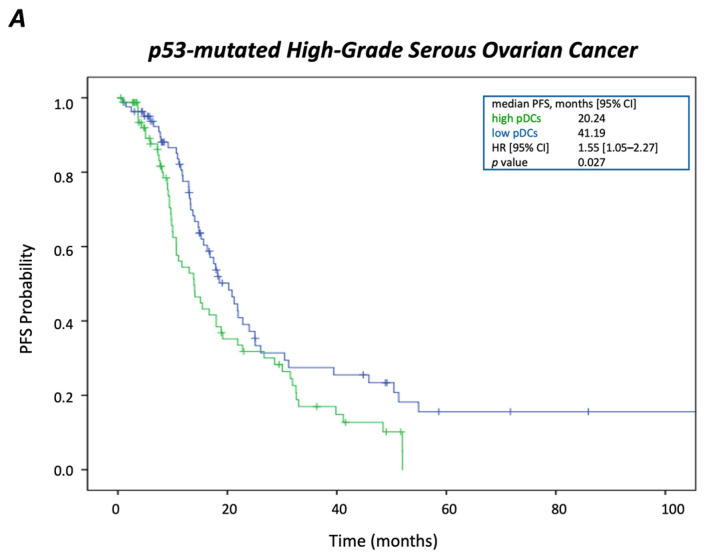

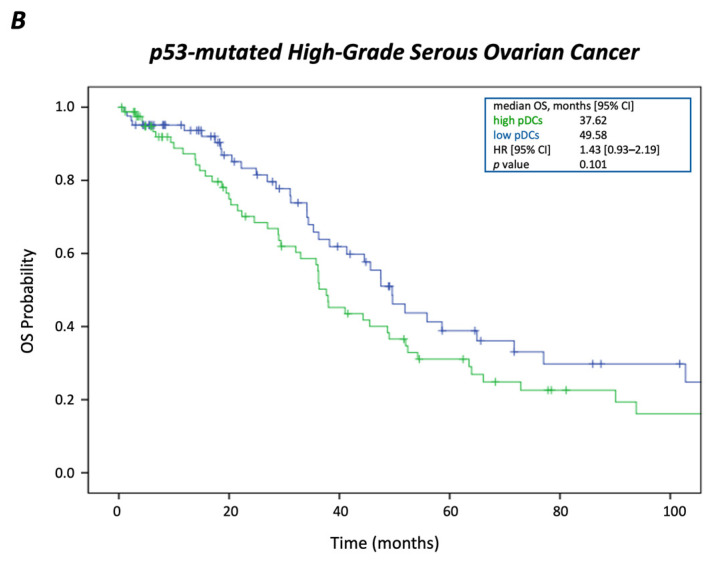
Prognostic impact of plasmacytoid dendritic cells in *TP53*-mutated high-grade serous ovarian cancer. Kaplan–Meier survival plots illustrating (**A**) progression-free survival and (**B**) overall survival in *TP53*-mutated high-grade serous ovarian cancer according to pDC abundance (high vs. low). Higher levels of pDCs were associated with shorter progression-free survival.

Despite the lower presence of pDCs in HGSOC compared to both other *TP53mut* tumor entities, when dichotomized along the median value into high and low abundance, high pDC infiltration revealed to be associated with impaired PFS (*p* = 0.027) in Kaplan–Meier analysis (Figure 2A). The independency of this finding was confirmed in the multivariable Cox regression analysis (Table 1).

However, as depicted in Figure 2B, no significant prognostic relevance in terms of OS was pointed out for the pDC subset. Importantly, neither in *TP53mut* EC nor in TNBC, the more amply represented pDC subsets, were found to affect clinical outcome. Likewise, none of the other analyzed DC subsets herein were pointed out to exhibit prognostic impact in any of the investigated tumor entities.

**Figure 3:**
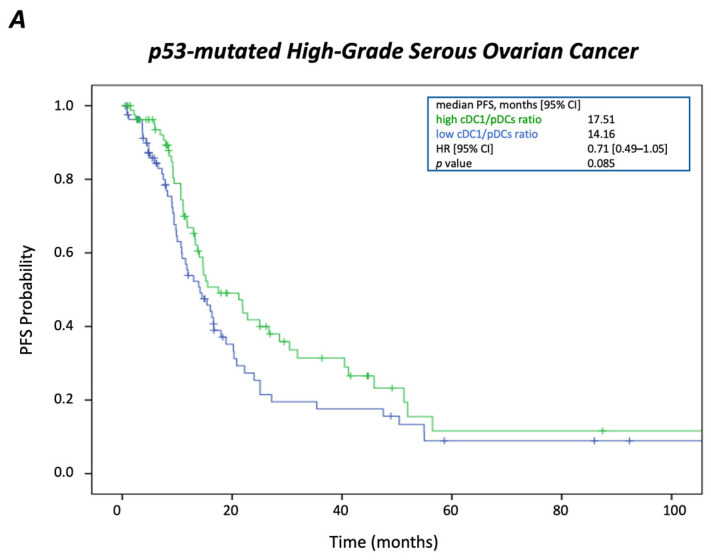

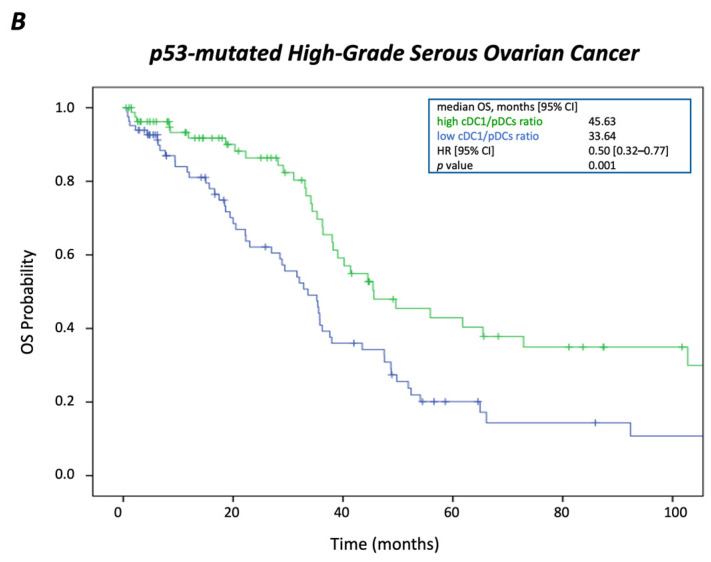
Prognostic impact of the cDC1/pDC ratio in *TP53*-mutated high-grade serous ovarian cancer. Kaplan–Meier survival plots showing (**A**) progression-free survival and (**B**) overall survival according to the cDC1/pDC ratio (high vs. low, median split). A higher cDC1/pDC ratio was significantly associated with prolonged overall survival.

When ratios of the prominent DC subsets were dichotomized along their respective median value, only patients with cancers exhibiting high cDC1/pDC ratio showed a longer median OS than those with low ratio (45.63 vs. 33.64 months; *p* = 0.001); however, no significant impact on PFS was observed for this DC relationship (Figure 3).

### 3.4. Tumor Mutational Burden and TP53 Mutation Characteristics

The assessed TMB was ultra-low in all the three *TP53mut* tumor entities, albeit the median TMB values differed significantly among the three tumor types. HGSOC exhibited the highest median TMB (2.10 Mut/Mb [IQR 1.50–3.00]), followed by TNBC (1.61 Mut/Mb [IQR 1.00–2.74]) and EC (1.27 Mut/Mb [IQR 0.92–1.82]) (*p* < 0.001). Of special note is that several DC subsets showed a significant inverse correlation with TMB in HGSOC, including cDC progenitor (r = −0.193, *p* = 0.014), cDC2 (r = −0.193, *p* = 0.014), and pDC (r = −0.172, *p* = 0.029). The full set of correlation results described in this section is provided in Supplementary Table S3.

**Figure 4:**
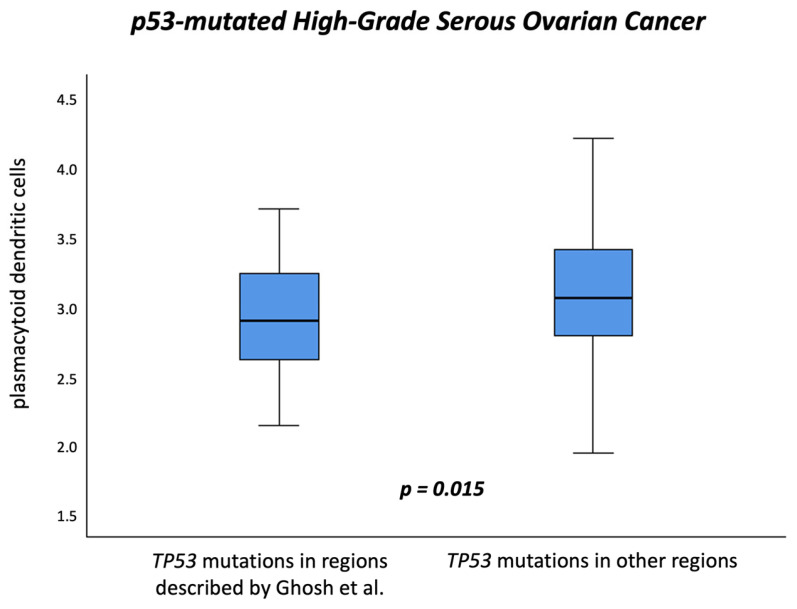
Association between *TP53* mutation localization and plasmacytoid dendritic cell abundance in *TP53*-mutated high-grade serous ovarian cancer. Boxplot showing pDC abundance in tumors with *TP53* mutations located in hotspot regions defined by Ghosh et al. [38] versus mutations in other regions. Tumors harboring *TP53* mutations in Ghosh-defined regions displayed significantly lower pDC abundance (median 2.89 vs. 3.05, *p* = 0.015).

To further explore whether the functional nature of *TP53* mutations contributes to the variability of TIME in HGSOC, all cases were classified GOF or LOF. LOF mutations predominated with 84.1%, (*n* = 269), while only 13.4% (*n* = 43) of cases harbored GOF mutations. However, no consistent associations were observed between the different *TP53* mutation classes and the abundance of the various DC subsets in the TIME, nor with the expression of key immunoregulatory genes such as *CXCL1*, *XBP1*, *FOXP3*, and *C1QA*, or IFN response scores in HGSOC. Further, when stratifying *TP53* aberrations either by mutation type or variant classification or functional domains, no significant association with pDC expression levels was uncovered (Table 2) However, when considering critical localizations of *TP53* mutations in specific hotspot regions, as proposed by Ghosh et al. [38], who recently demonstrated that specific mutant p53 forms suppress the signaling of the cGAS/STING pathway, differences in the pDC abundance were revealed. Tumors harboring *TP53* mutations in the Ghosh-defined hotspot regions displayed significantly lower pDC levels compared to mutations in all other regions (median 2.89 vs. 3.05, *p* = 0.015, Figure 4, Table 2). This finding was consistent when discriminating between the exact *TP53* aberrations analyzed by Ghosh et al., their proposed hotspot regions, and all other mutation positions (*p* = 0.027) [38]. In contrast, when analyzing cDC1 and cDC2 abundance in relation to *TP53* mutations located in the Ghosh-defined hotspot regions, no significant associations were observed.

### 3.5. Exploratory Analysis of TP53-Wildtype Tumors

Because *TP53* status was raised as a potential determinant of DC patterns, an exploratory post hoc comparison was conducted including all available *TP53*-wildtype (*TP53wt*) cases across EC, HGSOC, and TNBC. Across all three tumor entities, DC subset distributions did not differ meaningfully between *TP53wt* and *TP53mut* tumors. Furthermore, pDC abundance showed no association with PFS or OS in *TP53wt* HGSOC, EC, or TNBC (Supplementary Figure S1A–F).

## 4. Discussion

This study provides a detailed, cross-entity characterization of DC heterogeneity in *TP53mut* cancers, building on our previously published immunogenicity analysis of the same TCGA cohorts [13]. We now demonstrate that DC subset composition is highly entity-specific despite a shared *TP53mut* background and uniformly low TMB.

Notably, pDC abundance was highest in EC and TNBC compared to HGSOC. Intriguingly, however, only in HGSOC was pDC abundance of independent prognostic significance: high pDC values were associated with significantly shorter PFS. This suggests a tumor-type-specific anti-immunogenic and tolerogenic role of pDCs on its own, as no reliable associations were pointed out with most relevant immune modulatory factors and pathways, known to suppress anti-tumor immunity. Further, it supports prior observations that tumor-associated pDCs may acquire a tolerogenic phenotype that fosters tumor immune escape through Treg expansion, impaired type I IFN signaling, and altered metabolic states [44,45].

Notably, the prognostic effect of pDC abundance in *TP53mut* HGSOC was confined to PFS but not OS. This pattern, however, should be interpreted with caution. OS is a challenging endpoint for detecting immune-related prognostic markers in HGSOC, as it is heavily influenced by subsequent lines of therapy after first progression. Treatments frequently used in the recurrent setting—such as PARP inhibitors and anti-angiogenic agents—are known to modulate IFN-related pathways, DNA damage responses, and the composition of the TIME [46,47]. These therapy-induced alterations may mask baseline immune associations and thus attenuate the prognostic visibility of early TIME-derived features when using OS. In contrast, PFS might reflect the early interaction between tumor cells and the host immune system more accurately before these confounding influences accumulate. This provides a plausible explanation for why high pDC levels were associated with shorter PFS but not OS in our cohort. Importantly, this does not contradict the prognostic relevance observed for the cDC1/pDC ratio in OS analyses, as ratios rather capture the balance between immunostimulatory and tolerogenic DCs, which may remain informative despite treatment heterogeneity at later disease stages.

The consideration of the relationship between the functional (immune-stimulatory or -inhibiting) quality of the various DC subsets represented in the TIME revealed that only a high cDC1/pDC ratio was associated with improved OS. This may indicate that prognostic impairment of high pDC can be outperformed by cDC1 enrichment exceeding pDC abundance.

Importantly, our study challenges the simplistic assumption of a uniform link between the presence of DCs as specialized neoantigen presenting cells and an efficient anti-tumor immune response. TMB is often used as a surrogate marker for neoantigen load and immune ICI responsiveness [48]. Several DC subsets in this entity showed inverse correlations with TMB, including cDC2, cDC progenitor, and pDCs which all are rather promoting immune suppressive conditions. The observed inverse associations may indicate that, in the event of elevated neoantigen load, the presence of immune suppressive DCs might be reduced concomitantly for whatever reason but is leading toward a more immune amenable TIME.

In HGSOC only mature DCs demonstrated an inverse correlation with the IFN-α response, while all other subsets were unrelated to IFN-α and IFN-γ response. This may open a certain disconnection in the interplay between DCs and especially IFN-γ-driven pathways and may point to a broader dysfunction in the center stage for innate-adaptive immune regulation in the TIME of HGSOC. One possible explanation involves tumor-intrinsic mechanisms that actively suppress IFN responses, such as the downregulation of specific signaling pathways previously described in the literature [49]. Furthermore, there is evidence suggesting that DCs may undergo metabolic or transcriptional reprogramming within the TIME driven by TGF-β, prostaglandin E2, or Wnt signaling—all of which have been shown to influence DC maturation and impede IFN production [50,51,52].

The observed correlation between IFN-α response and M2 macrophage scores may reflect the complex interplay between type I IFN signaling and myeloid cell polarization in *TP53mut* HGSOC. While IFN-α is classically known for its antiviral and immunostimulatory functions, persistent or dysregulated type I IFN signaling has been implicated in immune exhaustion and tolerogenic circuitries in cancer. In HGSOC, pDCs are a key source of IFN-α and have been linked to immune suppression and an increased risk of early relapse [45]. This could be in line with the very recent findings of detrimental effects caused by a cytokine storm triggered by pDCs in antiviral innate immune response in mice [53]. Similarly, it is conceivable that pDCs are crucial in mounting excessive pro-inflammatory and tumor-promoting reactions in the TIME of HGSOC.

Nonetheless, in our cohort, pDC abundance showed no significant association with IFN-α response scores. Instead, pDCs correlated inversely with monocyte abundance and positively with neutrophils. The latter is frequently linked to M2-polarized macrophage infiltration [54,55]. This constellation suggests an immunologically hostile, inflammation-driven TIME, in which neutrophil-rich environments and altered myeloid cell composition contribute to tumor-promoting conditions. Although pDCs are a potential source of type I IFN, their prognostic impact in *TP53mut* HGSOC appears more closely related to myeloid cell interactions than to direct IFN-α–mediated effects. This is underscored by the lack of prognostic effects of IFN-α score in HGSOC.

Although increased pDC abundance could theoretically reflect a reactive infiltration pattern, several features of our data argue against a purely reactive influx. For example, pDC levels did not correlate with IFN-response scores, although such a correlation would be expected if pDC recruitment were predominantly driven by a classical type I IFN reaction. Nevertheless, because bulk RNA-seq cannot fully distinguish reactive recruitment from tumor-conditioned functional alterations, these interpretations should be considered with caution and remain context-dependent.

Given the prognostic relevance of pDCs in *TP53mut* HGSOC, we further explored whether their abundance was influenced by the underlying *TP53* mutation class. Beyond mutational subclassification, *TP53* is a central guardian of genome integrity, where intact p53 function supports genomic stability, apoptosis, and immune surveillance [1,56]. Its loss facilitates tumor development not only by enabling genomic instability but also by impairing antitumor immune responses, including antigen-presenting cells (APCs) recruitment and cytotoxic lymphocyte activation—mechanisms that are closely linked to the conceptual basis of cancer immunotherapy [33,38,57]. Previous studies have suggested that distinct *TP53* mutation classes may alter the TIME by modulating cytokine and chemokine profiles, which could affect immune cell recruitment and function [57]. While our results do not support the association between *TP53* functional class (GOF vs. LOF) and pDC infiltration, we did observe a significant link between critical mutation localization and pDC abundance. Specifically, HGSOC tumors harboring *TP53* mutations in the hotspot regions defined by Ghosh et al. displayed lower pDC levels compared to tumors with mutations in other regions [38]. Our findings therefore suggest that, at least with regard to pDCs, the localization of *TP53* mutations may modulate the immune contexture in HGSOC. Although correlative, this pattern aligns with insights from Ghosh et al., who demonstrated that several *TP53* hotspot mutants suppress cGAS–STING activation and thereby attenuate downstream type I IFN signaling [38]. As pDCs are major producers and responders of type I IFN, impaired innate sensing and IFN pathway activation in tumors carrying such hotspot mutations could plausibly contribute to reduced pDC abundance within the TIME. While our study cannot directly test this mechanism, the observed association is biologically consistent with mutation-specific disruption of innate immune signaling and supports the hypothesis that *TP53* mutation localization—rather than its functional class alone—may modulate DC composition in *TP53mut* HGSOC.

To evaluate whether *TP53* status itself could account for variation in DC abundance, we additionally examined *TP53wt* tumors across all three entities. *TP53wt* cases—rare in HGSOC and heterogeneous in EC and TNBC—showed no consistent differences in DC subset distribution, and pDC levels were not associated with survival outcomes. These exploratory findings indicate that the DC patterns observed in our study predominantly arise within the entity-specific immune environments characteristic of *TP53mut* tumors.

Taken together, these immune associations highlight that DCs are not only differentially abundant but obviously exhibit distinct cellular interaction profiles across various tumor entities, potentially shaping the immune contexture in a tumor-type-specific manner. Importantly, these findings expand upon the transcriptomic patterns and imply that the role of DCs in modulating immunity and tumor progression cannot be inferred from their mere presence alone. We show that DC subsets exhibit tumor-specific abundance patterns, immune correlations, and prognostic relevance across *TP53mut* EC, HGSOC, and TNBC with low TMB. Rather than uniformly promoting anti-tumor immunity, DCs may contribute to immune suppression in selected contexts. These findings align with the growing recognition that DCs, particularly in low-immunogenic tumors, may support immunosuppression under the influence of stromal or metabolic conditioning [32,58].

Given the limited therapeutic efficacy of ICIs in tumors with low TMB, such as the *TP53mut* cancers examined here, novel immunotherapeutic strategies are urgently needed. DCs representing central regulators of innate and adaptive immunity could represent an attractive target for immune modulation in these low-immunogenic tumors in future. This is further supported by ongoing clinical trials such as a recent phase I/II study in recurrent, platinum-resistant ovarian cancer (NCT05773859) [59] which is currently evaluating a neoantigen-pulsed autologous DC vaccine in combination with PD-1 blockade. This approach aims to enhance antitumor immune responses in low-immunogenic tumors, underscoring the need of translational research of DC-targeted strategies even in low-TMB malignancies such as HGSOC.

Finally, our findings highlight that bulk transcriptomic datasets may not be sufficient to capture the full complexity of DC function in tumors. Future studies using more refined analyses will be important to better understand how the various subsets of DCs interact exactly with other cells in the TIME—at least in low-immunogenic, *TP53mut* cancers. In addition to transcriptomic approaches, emerging experimental systems enabling selective pDC depletion (e.g., conditional pDC-knockout mouse models [53]) may provide valuable functional confirmation of the role of pDCs in early tumor-immune interactions. Therefore, this work is a first attempt to better understand the complexity in the interplay of the various DC subfractions within the onco-immune contexture. The exact knowledge of this network is an important prerequisite for all future endeavors focusing on the therapeutic targeting of DC subfractions in malignancies.

## 5. Conclusions

This study provides novel insight into the immune landscape of *TP53mut* cancers, with a primary focus on HGSOC. Among the three tumor types analyzed, HGSOC stood out by exhibiting a distinct DC profile, characterized by a lower abundance of pDCs but a unique prognostic impact: high pDC scores were independently associated with significantly shorter PFS. This suggests a tumor-specific, immune-suppressive role of pDCs that may contribute to disease progression in HGSOC. Moreover, in HGSOC, several immune-suppressive DC subsets showed inverse associations with TMB. None of the analyzed DC subsets were related to IFN-γ response and only mature DCs were inversely associated with IFN-α response score, and all this could point to a potential disconnection in the master function of DCs, namely the communication between innate and adaptive immune response within the TIME of HGSOC.

While this study is limited by its retrospective design and reliance on bulk transcriptomic datasets, it provides a solid foundation for research into the functional dynamics of DC subsets in *TP53mut* tumors. In malignancies with low TMB and poor responsiveness to ICIs, targeting DC subsets may represent a promising strategy to reprogram the TIME and enhance therapeutic efficacy of ICIs. This work represents a first step toward a more nuanced understanding of DC heterogeneity and its implications for immune escape in low-immunogenic tumors.

### Limitations

This study has some limitations that should be considered. All analyses were based on retrospective bulk RNA sequencing data from TCGA, which lacks spatial and functional resolution. As such, the use of transcriptomic DC signatures—though biologically informed—does not allow direct assessment of cell function, localization, or phenotype. Moreover, findings were not validated in an independent cohort, limiting generalizability. In addition, TCGA data originate from multiple centers and sequencing batches, and residual batch effects or dataset variability may influence transcriptomic estimates and cell-type deconvolution accuracy. Larger, prospective studies are needed to confirm the biological and therapeutic relevance of the observed patterns. Finally, survival analyses may be influenced by limited clinical annotation and cohort size. Despite these limitations, our study offers a comparative framework for understanding DC heterogeneity in *TP53mut* low-TMB tumors. This is an important prerequisite for the development and refinement of future DC-targeted immunotherapeutic strategies.

## Figures and Tables

**Table 1:** Results of univariate and multivariable Cox proportional hazards analysis for *TP53*-mutated high-grade serous ovarian cancer for various parameters including plasmacytoid dendritic cells (low vs. high).

		Univariate Analysis				Multivariable Analysis			
		PFS		OS		PFS		OS	
		HR [95%—CI]	*p*	HR [95%—CI]	*p*	HR [95%—CI]	*p*	HR [95%—CI]	*p*
***TP53*** **-mutated high-grade serous ovarian cancer**									
**Age**	low vs. high	1.31 [1–1.72]	**0.05**	1.53 [1.13–2.07]	**0.006**	1.69 [1.12–2.51]	**0.009**	2.21 [1.42–3.44]	**<0.** **001**
**FIGO stage**	I/II vs. III/IV	2.02 [1.04–3.94]	**0.039**	1.39 [0.65–2.97]	0.397	1.09 [0.44–2.68]	0.860	0.83 [0.30–2.29]	0.715
**Residual disease**	R0 vs. R1	0.87 [0.63–1.22]	0.427	0.87 [0.61–1.26]	0.464	0.88 [0.53–1.48]	0.639	0.84 [0.47–1.51]	0.554
**pDCs**	low vs. high	1.55 [1.05–2.27]	**0.027**	1.43 [0.93–2.19]	0.101	1.62 [1.09–2.40]	**0.017**	1.66 [1.06–2.59]	**0.026**

PFS, progression-free survival; OS, overall survival; FIGO stage, International Federation of Gynecology and Obstetrics stage; pDCs, plasmacytoid dendritic cells; significant *p*-values are indicated in bold.

**Table 2:** Association between *TP53* mutation localization (according to Ghosh et al.) and plasmacytoid dendritic cell abundance in *TP53*-mutated high-grade serous ovarian cancer (*n* = 162). Patients with multiple *TP53* mutations were excluded. Median enrichment score (GSVA) and median expression (log2 TPM+1) for pDC signatures are shown with ranges and corresponding *p*-values. Mutations in the Ghosh-defined regions were associated with significantly lower pDC abundance.

*TP53*-Mutated High-Grade Serous Ovarian Cancer					
	Number	Percentage	pDC		
			Median	Range	*p*-Value
**Variant type**					
SNP	129	80%	3.011	2.696–3.332	0.717
INS	7	4%	2.960	2.795–3.388	
DEL	26	16%	3.006	2.761–3.659	
**Variant classification**					
Missense	91	56%	3.040	2.761–3.356	0.161
Nonsense	19	12%	2.929	2.620–3.336	
Silent	1	1%	-	-	
Splice site	18	11%	2.744	2.598–3.200	
Frame shift indel	28	17%	2.970	2.760–3.377	
Inframe indel	5	3%	3.744	2.879–3.882	
3’ UTR	0	0%	-	-	
Intron	0	0%	-	-	
**Function**					
GOF	21	13%	3.022	2.961–3.200	0.565
LOF	141	87%	2.975	2.696–3.404	
**Localization (domains)**					
Transactivation TAD1	3	2%	3.752	-	0.209
Transactivation TAD2	3	2%	3.473	-	
SH3-like/Pro-rich	1	1%	-	-	
NA; N-term	1	1%	-	-	
DNA binding	136	84%	3.023	2.749–3.389	
NA; C-term	6	4%	2.792	2.520–2.955	
NLS	4	2%	3.024	2.937–3.544	
Tetramerization	8	5%	2.739	2.510–3.112	
Regulation	0	0%	-	-	
NA	0	0%	-	-	
***TP53* aberration in the relevant region described by Ghosh et al. [38]**					
(one patient with a silent mutation was excluded)					
No, other region	122	76%	3.050	2.775–3.406	**0.015**
Yes	39	24%	2.887	2.608–3.247	

Abbreviations: SNP, single nucleotide polymorphism; INS, insertion; DEL, deletion; GOF, gain-of-function; LOF, loss-of-function; NLS, nuclear localization signal; NA, not applicable. significant *p*-values are indicated in bold.

## Data Availability

Publicly available datasets were analyzed in this study. These data can be found at The Cancer Genome Atlas (TCGA) via the Genomic Data Commons (GDC) Data Portal: https://portal.gdc.cancer.gov/ (accessed on 5 August 2025).

## Supplementary Materials

The following supporting information can be downloaded at https://www.mdpi.com/article/10.3390/cancers17233877/s1; Table S1: Data sources used in this study; Table S2: Immune-related gene signatures; Table S3: Spearman correlation coefficients (r) and Bonferroni-adjusted *p*-values for associations between dendritic cell subsets and immune-related parameters in TP53-mutated high-grade serous ovarian cancer. Figure S1. Prognostic impact of plasmacytoid dendritic cells (pDCs) in TP53-wildtype tumors. References [60,61,62,63] are cited in the Supplementary Materials.

## Abbreviations

The following abbreviations are used in this manuscript:

APC	Antigen-presenting cells
BRCA1/2	Breast Cancer gene 1, Breast Cancer gene 2
cDC	Conventional dendritic cell
cDC1	Conventional dendritic cell type 1
cDC2	Conventional dendritic cell type 2
cGAS	Cyclic GMP-AMP synthase
CI	Confidence interval
C1QA	Complement component 1q subcomponent subunit A
CXCL1	C-X-C motif chemokine ligand 1
DC	Dendritic cell
EC	Endometrial cancer
ER	Estrogen receptor
FIGO	International Federation of Gynecology and Obstetrics
FOXP3	Forkhead box P3
GOF	Gain-of-function
GSVA	Gene set variation analysis
HGSOC	High-grade serous ovarian cancer
HER2	Human epidermal growth factor receptor 2
HR	Hazard ratio
ICI	Immune checkpoint inhibitor
IFN	Interferon
INS	Insertion
IQR	Interquartile range
LOF	Loss-of-function
MLH1	MutL homolog 1
Mb	Megabase
MSI	Microsatellite instability
NA	Not applicable
NK cell	Natural killer cell
NLS	Nuclear localization signal
OS	Overall survival
PD-1	Programmed cell death protein 1
PD-L1	Programmed death-ligand 1
pDC	Plasmacytoid dendritic cell
PFS	Progression-free survival
POLE	DNA polymerase epsilon
PR	Progesterone receptor
RNA	Ribonucleic Acid
RSEM	RNA-Seq by Expectation Maximization
SNP	Single nucleotide polymorphism
STING	Stimulator of interferon genes
TCGA	The Cancer Genome Atlas
TGF-β	Transforming growth factor beta
TIME	Tumor immune microenvironment
TMB	Tumor mutational burden
TNBC	Triple-negative breast cancer
TP53	Tumor protein p53 gene
TP53mut	TP53-mutated
TPM	Transcripts per million
Treg	Regulatory T cell
Wnt	Wingless/Integrated signaling pathway
XBP1	X-box binding protein 1
