# Supplementary material for: The Role of Plasmacytoid Dendritic Cells in the Immune Contexture of TP53-Mutated High-Grade Serous Ovarian Cancer
Source: Cancers (Basel). 2025 Dec 3;17(23):3877. doi: 10.3390/cancers17233877 (PMC12691120; doi:10.3390/cancers17233877)
Supplement: Supplementary file 1 [file cancers-17-03877-s001.zip › cancers-3979482-supplementary.pdf]

Supplementary Tables

Table S1: Data sources used in this study

Data	File	URL	Ref.
Tumor mutational burden (TMB)	Mutation-load-updated.txt	https://gdc.cancer.gov/about-data/publications/PanCan-CellOfOrigin	[60]
Somatic mutations (MC3)	mc3.v0.2.8.PUBLIC.maf.gz	https://gdc.cancer.gov/about-data/publications/mc3-2017	[35]
Survival information (TCGA-CDR)	Table S1<br>1-s2.0-S0092867418302290-mmc1.xlsx	https://doi.org/10.1016/j.cell.2018.02.052	[61]
Clinical data	UCEC:Clinical_Pick_Tier1<br>OV: Clinical_Pick_Tier1<br>BRCA: Clinical_Pick_Tier1, Merge_Clinical	http://firebrowse.org/	[18]<br>[19]<br>[17]
Microsatellite instability (MSI) status	Table S4<br>1-s2.0-S1535610818301193-mmc4	https://doi.org/10.1016/j.ccell.2018.03.014	[36]
RNA seq V2 (RSEM)	UCEC: illuminahiseq_rnaseqv2-RSEM_genes<br>UCEC: illuminaga_rnaseqv2-RSEM_genes<br>OV: illuminahiseq_rnaseqv2-RSEM_genes<br>BRCA: illuminahiseq_rnaseqv2-RSEM_genes	http://firebrowse.org/	[18]<br>[19]<br>[17]

Table S2: Immune-related gene signatures

SIGNATURE	HUGO GENE SYMBOLS	URL	Ref.
IFNG signature	*IFNG, STAT1, CCR5, CXCL9, CXCL10, CXCL11, IDO1, PRF1, GZMA, MHCII HLA-DRA*	https://doi.org/10.1172/JCI91190	[62]
Interferon Alpha Response	*MX1, ISG15, OAS1, IFIT3, IFI44, IFI35, IRF7, RSAD2, IFI44L, IFITM1, IFI27, IRF9, OASL, EIF2AK2, IFIT2, CXCL10, TAP1, SP110, DDX60, UBE2L6, USP18, PSMB8, IFIH1, BST2, LGALS3BP, ADAR, ISG20, GBP2, IRF1, PLSCR1, PSMB9, HERC6, SAMD9, CMPK2, IFITM3, RTP4, STAT2, SAMD9L, LY6E, IFITM2, HELZ2, CXCL11, TRIM21, PARP14, TRIM26, PARP12, NMI, RNF31, HLA-C, CASP1, TRIM14, TDRD7, DHX58, PARP9, PNPT1, TRIM25, PSME1, WARS1, EPSTI1, UBA7, PSME2, B2M, TRIM5, C1S, LAP3, LAMP3, GBP4, NCOA7, TMEM140, CD74, GMPR, PSMA3, PROCR, IL7, IFI30, IRF2, CSF1, IL15, CNP, TENT5A, IL4R, CMTR1, CD47, LPAR6, MOV10, CASP8, TXNIP, SLC25A28, SELL, TRAFD1, BATF2, RIPK2, CCRL2, NUB1, OGFR, MVB12A, ELF1*	http://www.gsea-msigdb.org/gsea/msigdb/cards/HALLMARK_INTERFERON_ALPHA_RESPONSE	[63]
Interferon Gamma Response	*STAT1, ISG15, IFIT1, MX1, IFIT3, IFI35, IRF7, IFIT2, OAS2, TAP1, EIF2AK2, RSAD2, MX2, IRF1, OAS3, TNFSF10, IRF9, CXCL10, IFI44, BST2, XAF1, SP110, OASL, PSMB8, IFI44L, IFITM3, DDX60, LGALS3BP, GBP4, IRF8, PSMB9, PML, IFIH1, UBE2L6, IFI27, ADAR, LY6E, STAT2, CXCL9, IL10RA, PLA2G4A, TRIM21, USP18, PTGS2, EPSTI1, C1S, DDX58, IL15, NLRC5, NMI, IDO1, PSMB10, CXCL11, ITGB7, SAMHD1, HERC6, CMPK2, SAMD9L, RTP4, PTPN2, PARP14, TNFAIP2, IFITM2, PLSCR1, SOCS1, CASP1, ICAM1, WARS1, PSME1, ISG20, IRF2, TRIM14, FCGR1A, MARCHF1, SOCS3, JAK2, HLA-DMA, PARP12, TNFAIP6, TRIM26, VCAM1, CD274, CIITA, NAMPT, SELP, GPR18, FPR1, HELZ2, PSME2, SERPING1, CCL5, RNF31, SOD2, TRIM25, LAP3, PSMA3, RNF213, PELI1, CFB, CD86, TXNIP, HLA-DQA1, GCH1, PNP, CCL7, PTPN6, SPPL2A, IL4R,*	http://www.gsea-msigdb.org/gsea/msigdb/cards/HALLMARK_INTERFERON_GAMMA_RESPONSE	[63]

	PNPT1, DHX58, BTG1, CASP8, IFI30, CCL2, FGL2, CASP7, SECTM1, IL15RA, CD40, TRAFD1, HLA-DRB1, GBP6, LCP2, HLA-G, MT2A, RIPK1, KLRK1, UPP1, PSMB2, TDRD7, HIF1A, EIF4E3, VAMP8, PFKP, CD38, ZBP1, BANK1, TOR1B, RBCK1, PDE4B, MVP, IL7, BPGM, CMTR1, AUTS2, B2M, RIPK2, CD69, MYD88, PSMA2, PIM1, NOD1, CFH, TAPBP, SLC25A28, PTPN1, TNFAIP3, SSPN, NUP93, MTHFD2, CDKN1A, IRF4, NFKB1, BATF2, HLA-B, LATS2, IRF5, SLAMF7, ISOC1, P2RY14, STAT3, NCOA3, HLA-A, IL6, GZMA, IFNAR2, CD74, RAPGEF6, CASP4, FAS, OGFR, ARL4A, SRI, LYSMD2, CSF2RB, ST3GAL5, C1R, CASP3, CMKLR1, NFKBIA, METTL7B, ST8SIA4, XCL1, IL2RB, VAMP5, IL18BP, ZNFX1, ARID5B, APOL6, STAT4		
cDC progenitor	*AXL, MRC1*	https://doi.org/10.1101/2024.08.26.609563	[41]
cDC1	*CLEC9A, XCR1, CLNK, CADM1,WDFY4*	https://doi.org/10.1101/2024.08.26.609563	[41]
cDC2	*CD1C, CD1E, CLEC10A, FCER1A*	https://doi.org/10.1101/2024.08.26.609563	[41]
pDC	*IL3RA, ITM2C, SMPD3, TCF4, BLNK*	https://doi.org/10.1101/2024.08.26.609563	[41]
Mature DC	*CCL22, CCR7, LAMP3*	ttps://doi.org/10.1101/2024.08.26.609563	[41]

Table S3: Spearman correlation coefficients (r) and Bonferroni-adjusted p-values for associations between dendritic cell subsets and immune-related parameters in *TP53*-mutated high-grade serous ovarian cancer.

		cDC progenitor	cDC1	cDC2	pDC	mature DC	IFN-α response	IFN-γ response	M2-like macrophages	monocytes	neutrophils	CD8+ T cells	CD4+ T cells	Tregs	B cells	NK cells	M1-like macrophages	*CD274* (PDL1)	*C1QA*	*FOXP3*	*XBP1*	*CXCL10*	TMB
**cDC progenitor**	r	-	.517	.527	.464	.335	-0.136	-0.078	0.086	-0.034	0.108	0.003	-0.047	-0.074	0.049	0.119	-0.103	0.108	0.035	0.024	-0.04	-0.036	-.193
	p	-	0	0	0	0	0.202	0.465	0.411	0.664	0.169	0.979	0.657	0.48	0.639	0.256	0.325	0.301	0.738	0.818	0.705	0.729	**0.014**
**cDC1**	r	.517	-	.467	.371	.291	-0.002	0.05	-0.037	0.048	0.144	0.117	-0.111	0.074	-0.012	0.081	0.046	0.125	0.011	0.045	-0.113	-0.023	-0.092
	p	0	-	0	0	0	0.982	0.638	0.723	0.541	0.066	0.264	0.29	0.484	0.907	0.439	0.66	0.233	0.92	0.669	0.279	0.826	0.246
**cDC2**	r	.527	.467	-	.224	.289	-0.159	-0.148	0.086	0.148	-0.05	0.091	-0.034	-0.016	0.042	0.101	-0.114	0.055	-0.021	0.05	0.054	-0.085	-.193
	p	0	0	-	**0.004**	0	0.135	0.165	0.411	0.058	0.52	0.384	0.745	0.879	0.691	0.335	0.276	0.597	0.844	0.631	0.606	0.419	**0.014**
**pDC**	r	.464	.371	.224	-	0.132	0.002	-0.022	0.019	-.209	.171	-0.091	0.091	-0.071	-0.018	0.075	-.279	-0.019	-0.17	-0.113	-0.001	-0.149	-.172
	p	0	0	**0.004**	-	0.091	0.982	0.84	0.856	**0.007**	**0.028**	0.384	0.387	0.496	0.862	0.477	**0.007**	0.856	0.104	0.282	0.994	0.154	**0.029**
**mature DC**	r	.335	.291	.289	0.132	-	-.238	-0.185	-0.004	0.024	0.036	-0.012	0.089	0.039	0.085	-0.113	-0.063	0.095	-0.015	0.063	0.074	-0.006	-0.072
	p	0	0	0	0.091	-	**0.024**	0.081	0.967	0.757	0.649	0.907	0.395	0.712	0.42	0.282	0.55	0.367	0.884	0.55	0.479	0.952	0.365
**IFN-α response**	r	-0.136	-0.002	-0.159	0.002	-.238	-	.936	.207	0.182	-0.134	.313	-0.063	.207	0.02	-0.06	.326	.513	.387	.330	0.126	.580	0.002
	p	0.202	0.982	0.135	0.982	**0.024**	-	0	**0.014**	0.087	0.208	0	0.46	**0.015**	0.815	0.48	0	0	0	0	0.141	0	0.978
**IFN-γ response**	r	-0.078	0.05	-0.148	-0.022	-0.185	.936	-	.244	0.165	-0.112	.444	-0.112	.302	0.082	-0.055	.467	.561	.539	.459	.179	.612	0.075
	p	0.465	0.638	0.165	0.84	0.081	0	-	**0.004**	0.121	0.293	0	0.19	0	0.339	0.518	0	0	0	0	**0.035**	0	0.342
**M2-like macrophages**	r	0.086	-0.037	0.086	0.019	-0.004	.207	.244	-	0.034	0.007	0.134	-.261	.364	0.147	0.113	.270	.414	.471	.308	0.073	.308	0.046
	p	0.411	0.723	0.411	0.856	0.967	**0.014**	**0.004**	-	0.749	0.948	0.085	**0.001**	0	0.059	0.15	0	0	0	0	0.355	0	0.563
**monocytes**	r	-0.034	0.048	0.148	-.209	0.024	0.182	0.165	0.034	-	-.668	0.013	0.114	0.098	-0.147	0.108	0.033	0.094	0.058	0.108	-0.059	0.129	-0.073
	p	0.664	0.541	0.058	**0.007**	0.757	0.087	0.121	0.749	-	0	0.9	0.276	0.35	0.158	0.303	0.752	0.37	0.581	0.304	0.574	0.219	0.359
**neutrophils**	r	0.108	0.144	-0.05	.171	0.036	-0.134	-0.112	0.007	-.668	-	0.068	-0.015	-0.063	-0.088	-.277	0.09	0.006	0.002	-0.105	0.146	0.003	0.066
	p	0.169	0.066	0.52	**0.028**	0.649	0.208	0.293	0.948	0	-	0.517	0.883	0.549	0.4	**0.007**	0.393	0.957	0.985	0.317	0.164	0.976	0.404

**CD8+ T cells**	r	0.003	0.117	0.091	-0.091	-0.012	.313	.444	0.134	0.013	0.068	-	-0.147	.344	0.06	-0.103	.361	.447	.509	.589	.242	.494	0.154
	p	0.979	0.264	0.384	0.384	0.907	0	0	0.085	0.9	0.517	-	0.059	0	0.446	0.189	0	0	0	0	**0.002**	0	0.051
**CD4+ T cells**	r	-0.047	-0.111	-0.034	0.091	0.089	-0.063	-0.112	-.261	0.114	-0.015	-0.147	-	-.553	-.497	-.333	-.329	-.277	-.172	-.237	0.107	-0.113	0.041
	p	0.657	0.29	0.745	0.387	0.395	0.46	0.19	**0.001**	0.276	0.883	0.059	-	0	0	0	0	0	**0.027**	**0.002**	0.17	0.15	0.603
**Tregs**	r	-0.074	0.074	-0.016	-0.071	0.039	.207	.302	.364	0.098	-0.063	.344	-.553	-	.278	.230	.472	.425	.366	.524	0.136	.298	0.063
	p	0.48	0.484	0.879	0.496	0.712	**0.015**	0	0	0.35	0.549	0	0	-	0	**0.003**	0	0	0	0	0.082	0	0.425
**B cells**	r	0.049	-0.012	0.042	-0.018	0.085	0.02	0.082	0.147	-0.147	-0.088	0.06	-.497	.278	-	0.105	.182	.309	0.141	.209	-0.074	0.087	0
	p	0.639	0.907	0.691	0.862	0.42	0.815	0.339	0.059	0.158	0.4	0.446	0	0	-	0.181	**0.019**	0	0.07	**0.007**	0.343	0.267	1
**NK cells**	r	0.119	0.081	0.101	0.075	-0.113	-0.06	-0.055	0.113	0.108	-.277	-0.103	-.333	.230	0.105	-	0.024	0.074	-0.062	0.014	-.204	-.200	0.001
	p	0.256	0.439	0.335	0.477	0.282	0.48	0.518	0.15	0.303	**0.007**	0.189	0	**0.003**	0.181	-	0.764	0.348	0.431	0.858	**0.009**	**0.01**	0.989
**M1-like macrophages**	r	-0.103	0.046	-0.114	-.279	-0.063	.326	.467	.270	0.033	0.09	.361	-.329	.472	.182	0.024	-	.420	.592	.457	0.054	.416	0.093
	p	0.325	0.66	0.276	**0.007**	0.55	0	0	0	0.752	0.393	0	0	0	**0.019**	0.764	-	0	0	0	0.489	0	0.24
***CD274* (PDL1)**	r	0.108	0.125	0.055	-0.019	0.095	.513	.561	.414	0.094	0.006	.447	-.277	.425	.309	0.074	.420	-	.512	.580	.196	.597	0.038
	p	0.301	0.233	0.597	0.856	0.367	0	0	0	0.37	0.957	0	0	0	0	0.348	0	-	0	0	**0.012**	0	0.628
C1QA	r	0.035	0.011	-0.021	-0.17	-0.015	.387	.539	.471	0.058	0.002	.509	-.172	.366	0.141	-0.062	.592	.512	-	.603	0.15	.624	.250
	p	0.738	0.92	0.844	0.104	0.884	0	0	0	0.581	0.985	0	**0.027**	0	0.07	0.431	0	0	-	0	0.054	0	**0.001**
FOXP3	r	0.024	0.045	0.05	-0.113	0.063	.330	.459	.308	0.108	-0.105	.589	-.237	.524	.209	0.014	.457	.580	.603	-	.196	.573	0.146
	p	0.818	0.669	0.631	0.282	0.55	0	0	0	0.304	0.317	0	**0.002**	0	**0.007**	0.858	0	0	0	-	**0.012**	0	0.064
XBP1	r	-0.04	-0.113	0.054	-0.001	0.074	0.126	.179	0.073	-0.059	0.146	.242	0.107	0.136	-0.074	-.204	0.054	.196	0.15	.196	-	.265	.261
	p	0.705	0.279	0.606	0.994	0.479	0.141	**0.035**	0.355	0.574	0.164	**0.002**	0.17	0.082	0.343	**0.009**	0.489	**0.012**	0.054	**0.012**	-	**0.001**	**0.001**
CXCL10	r	-0.036	-0.023	-0.085	-0.149	-0.006	.580	.612	.308	0.129	0.003	.494	-0.113	.298	0.087	-.200	.416	.597	.624	.573	.265	-	.206
	p	0.729	0.826	0.419	0.154	0.952	0	0	0	0.219	0.976	0	0.15	0	0.267	**0.01**	0	0	0	0	**0.001**	-	**0.008**
**TMB**	r	-.193	-0.092	-.193	-.172	-0.072	0.002	0.075	0.046	-0.073	0.066	0.154	0.041	0.063	0	0.001	0.093	0.038	.250	0.146	.261	.206	-
	p	**0.014**	0.246	**0.014**	**0.029**	0.365	0.978	0.342	0.563	0.359	0.404	0.051	0.603	0.425	1	0.989	0.24	0.628	**0.001**	0.064	**0.001**	**0.008**	-

Supplementary Figures

A

TP53wt High-Grade Serous Ovarian Cancer

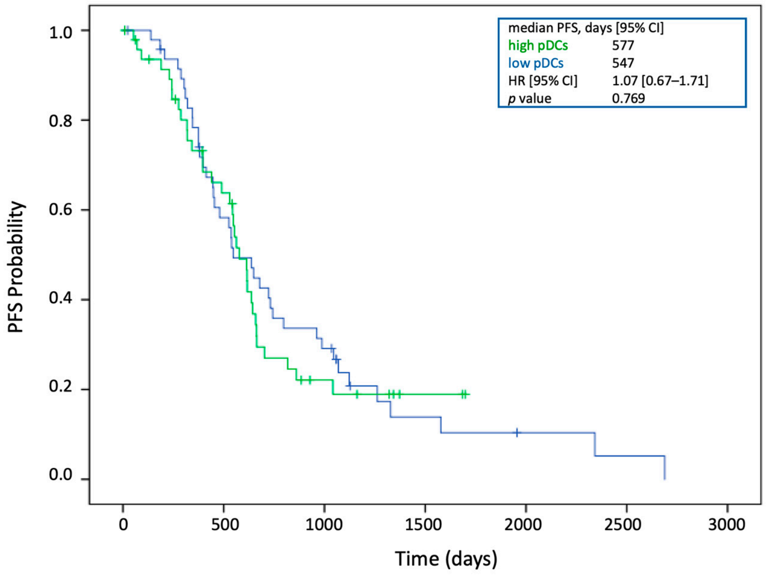

B

TP53wt High-Grade Serous Ovarian Cancer

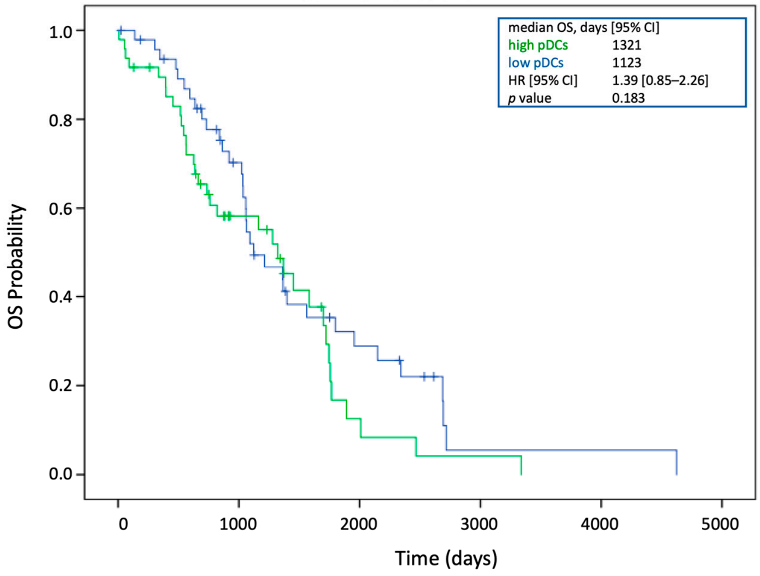

C

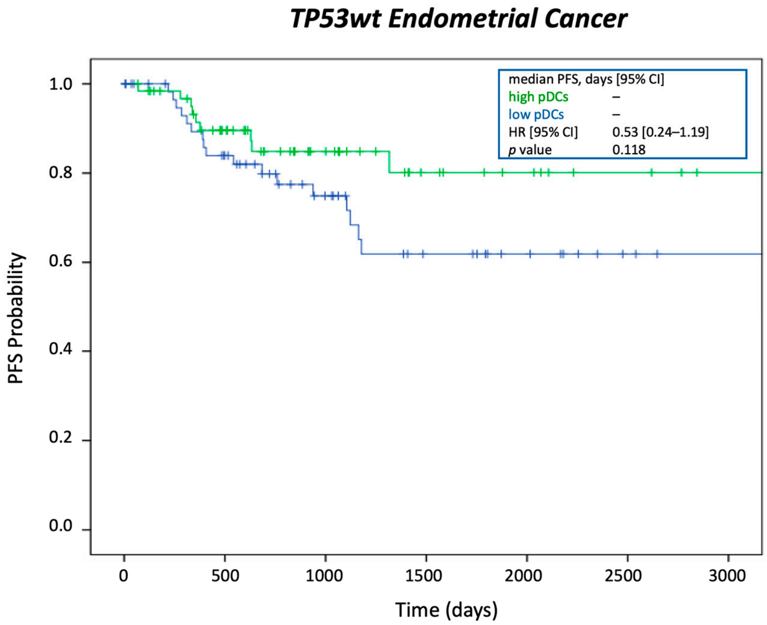
TP53wt Endometrial Cancer
median PFS, days [95% CI]
high pDCs –
low pDCs –
HR [95% CI] 0.53 [0.24–1.19]
p value 0.118
PFS Probability
1.0
0.8
0.6
0.4
0.2
0.0
0
500
1000
1500
2000
2500
3000
Time (days)

D

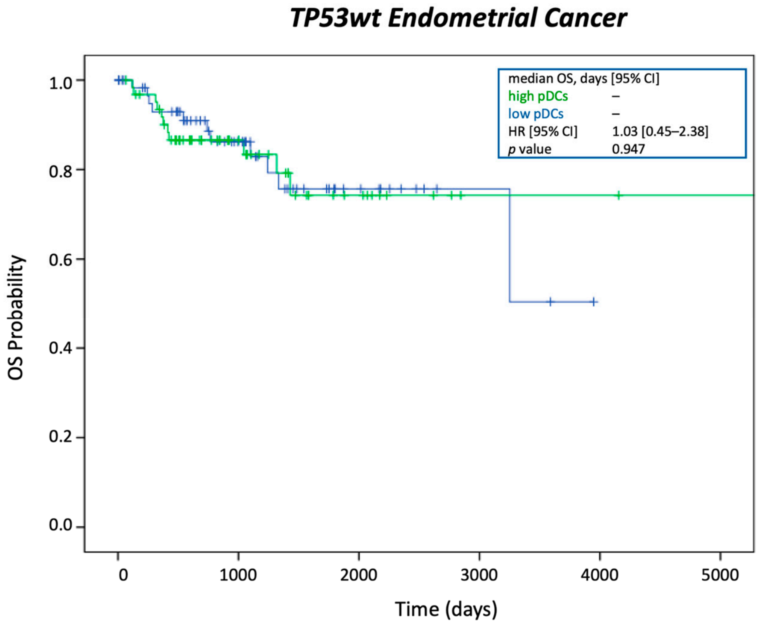
TP53wt Endometrial Cancer
median OS, days [95% CI]
high pDCs –
low pDCs –
HR [95% CI] 1.03 [0.45–2.38]
p value 0.947
OS Probability
1.0
0.8
0.6
0.4
0.2
0.0
0
1000
2000
3000
4000
5000
Time (days)

E

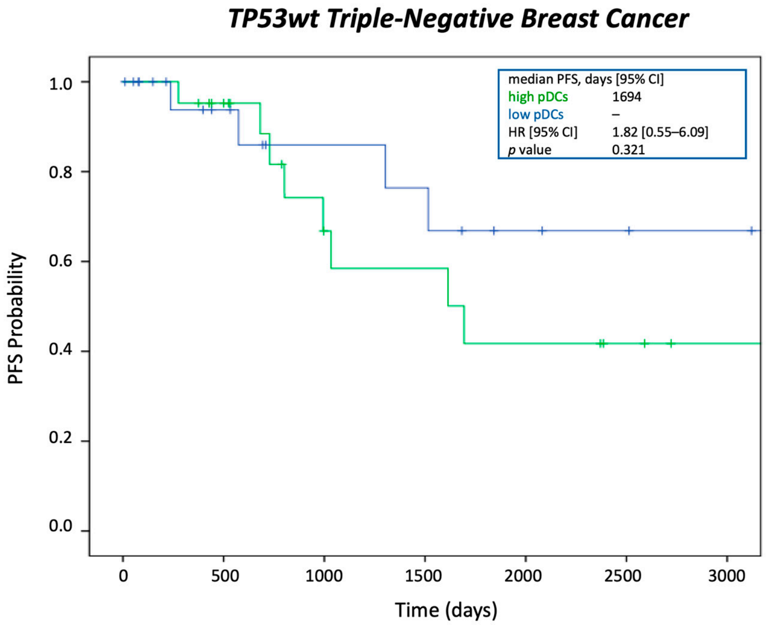

F

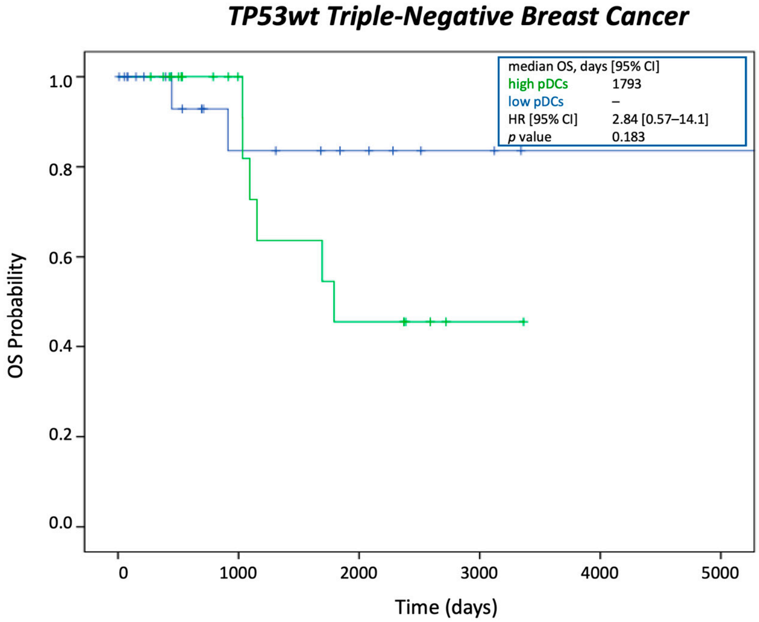

Supplementary Figure S1. Prognostic impact of plasmacytoid dendritic cells (pDCs) in TP53-wildtype tumors.

(A, B) – Kapan–Meier survival curves illustrating (A) progression-free survival and (B) overall survival in TP53-wildtype HGSOC according to pDC abundance (high vs. low). No significant association was observed between pDC levels and either PFS or OS in TP53wt HGSOC.
(C, D) – Kaplan–Meier survival curves showing (C) progression-free survival and (D) overall survival in TP53-wildtype endometrial cancer stratified by pDC abundance. pDC levels showed no prognostic relevance in TP53wt EC.
(E, F) – Kaplan–Meier survival curves showing (E) progression-free survival and (F) overall survival in TP53-wildtype TNBC stratified by pDC abundance. No significant differences in PFS or OS were observed.

References

17. Cancer Genome Atlas Network; Koboldt, D.C.; Fulton, R.S.; McLellan, M.D.; Schmidt, H.; Kalicki-Veizer, J.; McMichael, J.F.; Fulton, L.L.; Dooling, D.J.; Ding, L.; et al. Comprehensive molecular portraits of human breast tumours. *Nature* **2012**, *490*, 61–70.
18. Kandoth, C.; Schultz, N.; Cherniack, A.D.; Akbani, R.; Liu, Y.; Shen, H.; Robertson, A.G.; Pashtan, I.; Shen, R.; Benz, C.C.; et al. Integrated genomic characterization of endometrial carcinoma. *Nature* **2013**, *497*, 67–73.
19. Cancer Genome Atlas Research Network; Bell, D.; Berchuck, A.; Birrer, M.; Chien, J.; Cramer, D.W.; Dao, F.; Dhir, R.; Disaia, P.; Gabra, H.; et al. Integrated genomic analyses of ovarian carcinoma. *Nature* **2011**, *474*, 609–615.
35. Ellrott, K.; Bailey, M.H.; Saksena, G.; Covington, K.R.; Kandoth, C.; Stewart, C.; Hess, J.; Ma, S.; Chiotti, K.E.; McLellan, M.D.; et al. Scalable Open Science Approach for Mutation Calling of Tumor Exomes Using Multiple Genomic Pipelines. *Cell Syst.* **2018**, *6*, 271–281.e7.
36. Berger, A.C.; Korkut, A.; Kanchi, R.S.; Hegde, A.M.; Lenoir, W.; Liu, W.; Liu, Y.; Fan, H.; Shen, H.; Ravikumar, V.; et al. A Comprehensive Pan-Cancer Molecular Study of Gynecologic and Breast Cancers. *Cancer Cell* **2018**, *33*, 690–705.e9.
41. Marteau, V.; Nemati, N.; Handler, K.; Raju, D.; Kirchmair, A.; Rieder, D.; Nemethova, V.; Meier, C.; Leitner, J.; Pichler, V.; et al. Single-cell integration and multi-modal profiling reveals phenotypes and spatial organization of neutrophils in colorectal cancer. *bioRxiv*, **2025**, bioRxiv:2024.08.26.609563.
60. Hoadley, K.A.; Yau, C.; Hinoue, T.; Wolf, D.M.; Lazar, A.J.; Drill, E.; Shen, R.; Taylor, A.M.; Cherniack, A.D.; Thorsson, V.; et al. Cell-of-Origin Patterns Dominate the Molecular Classification of 10,000 Tumors from 33 Types of Cancer. *Cell* **2018**, *173*, 291–304.e6.
61. Liu, J.; Lichtenberg, T.; Hoadley, K.A.; Poisson, L.M.; Lazar, A.J.; Cherniack, A.D.; Kovatich, A.J.; Benz, C.C.; Levine, D.A.; Lee, A.V.; et al. An Integrated TCGA Pan-Cancer Clinical Data Resource to Drive High-Quality Survival Outcome Analytics. *Cell* **2018**, *173*, 400–416.e11.
62. Ayers, M.; Lunceford, J.; Nebozhyn, M.; Murphy, E.; Loboda, A.; Kaufman, D.R.; Albright, A.; Cheng, J.D.; Kang, S.P.; Shankaran, V.; et al. IFN-γ-related mRNA profile predicts clinical response to PD-1 blockade. *J. Clin. Investig.* **2017**, *127*, 2930–2940.
63. Liberzon, A.; Birger, C.; Thorvaldsdóttir, H.; Ghandi, M.; Mesirov, J.P.; Tamayo, P.; Parker, J.; Liu, J.; Zhang, Y.; Wong, C.; et al. The Molecular Signatures Database (MSigDB) hallmark gene set collection. *Cell Syst.* **2015**, *1*, 417–425.
